# Vision-Related Quality of Life in Patients With Rhegmatogenous Retinal Detachment Treated With Pars-Plana Vitrectomy: Impact of Gas Tamponade

**DOI:** 10.7759/cureus.38969

**Published:** 2023-05-13

**Authors:** Genovefa Machairoudia, Dimitrios Kazantzis, Irini Chatziralli, Georgios Theodossiadis, Ilias Georgalas, Panagiotis Theodossiadis

**Affiliations:** 1 Second Department of Ophthalmology, National and Kapodistrian University of Athens School of Medicine, Athens, GRC

**Keywords:** gas tamponade, sulfur hexafluoride, perfluoropropane, vision-related quality of life, pars plana vitrectomy, retinal detachment (rd)

## Abstract

Purpose: To investigate changes in vision-related quality of life in patients treated with pars plana vitrectomy (PPV) for rhegmatogenous retinal detachment (RRD) and compare groups according to the type of gas tamponade used.

Methods: Participants in this study were 48 patients with RRD who were treated with PPV and gas tamponade (sulfur hexafluoride (SF_6_) or perfluoropropane (C_3_F_8_)) without internal limiting membrane peeling. All participants underwent slit-lamp examination, fundoscopy, axial-length measurement, and completed the Vision Function Questionnaire-25 (VFQ-25) at month six postoperatively. We compared VFQ-25 composite and subscale scores in the SF_6_ and C_3_F_8_ groups and investigated any correlations between age, best corrected visual acuity (BCVA), axial length, and VFQ-25 scores.

Results: The demographic and clinical characteristics of the two groups (axial length, macular status, retinal detachment extent, duration of symptoms, and lens status) were comparable between the two groups. We found a statistically significant decrease in general vision (GV), ocular pain (OP), and driving (D) scores in the C_3_F_8_ group compared to the SF_6_ group. The VFQ-25 composite score was comparable in the two groups. Similarly, all other subscales of the VFQ-25 did not differ significantly between the two groups. Age and BCVA did not significantly correlate with VFQ-25 composite and subscale scores.

Conclusion: Specific VFQ-25 subscales were decreased in patients with RRD treated with C_3_F_8_ as a gas tamponade compared to SF_6_. This finding warrants further research in the tamponade agents used in PPV surgeries.

## Introduction

Rhegmatogenous retinal detachment (RRD) is caused by a retinal break, which allows fluid to accumulate in the space between the neurosensory retina and the retinal pigment epithelium. Although scleral buckling and pneumatic retinopexy have been proven to be efficacious in the treatment of RRD, pars plana vitrectomy (PPV) has emerged as the gold standard of RRD repair with primary repair rates of more than 90% [1,2]. Both silicone oil and gas tamponades have been used as tamponade agents in PPV for RRD repair with good functional and anatomical results [3]; however, functional results are often highly variable [4]. In case where gas is used as a tamponade agent, many parameters have been associated with the final visual outcome after PPV for RRD repair, including retinal detachment extent, macular involvement, duration of symptoms, lens status, use of perfluorocarbon liquid, and type of gas tamponade used [5-8].

Previous studies have tried to assess the vision-related quality of life in RRD patients by using vision-specific health-related quality of life questionnaires to investigate functional outcomes in patients with RRD treated with PPV [9-11]. One such questionnaire is the National Eye Institute Visual Function Questionnaire-25 (NEI-VFQ-25), a 25-item questionnaire that is widely used to assess vision-related quality of life in a variety of ocular diseases, such as age-related macular degeneration, cataract, diabetic retinopathy, and keratoconus [12-16]. The VFQ-25 encompasses 12 sub-scales and a composite score and has been validated in many different populations for a variety of diseases [17,18].

The purpose of this study was to examine the effect of gas tamponade agents on the vision-related quality of life in patients with RRD treated with PPV. We also aimed to investigate any correlations between age, best-corrected visual acuity, axial length, and the VFQ-25 composite and sub-scale scores.

## Materials and methods

This prospective non-randomized study was conducted at the Department of Ophthalmology, National and Kapodistrian University of Athens, Athens, Greece. The study was approved by the Institutional Review Board of “Attikon” General Hospital (121/5-3-2020) and was conducted in accordance with the Tenets of the Declaration of Helsinki. Written informed consent was obtained from all participants.

Retinal detachment was diagnosed by indirect dilated fundoscopy and confirmed with a B-scan in all cases. The demographic characteristics of patients (age and gender), the lens status, and the axial length and specific characteristics of RRD, i.e., the macula status (on/off), the extent of the RRD and the approximate duration of RRD were recorded for each patient preoperatively.

All patients underwent 23G PPV using the Constellation Vision System (Alcon Laboratories, Inc., Fort Worth, TX, USA) and gas tamponade with 20% sulfur hexafluoride (SF6) or 14% perfluoropropane (C_3_F_8_), without internal limiting membrane (ILM) peeling. The criteria used for the endotamponade selection were the intraocular pathological conditions, such as tear size, number, location, and detachment extent. The intraocular pressure (IOP) during surgery was set to 20-25 mmHg based on the specific settings of the machine. Combined cataract surgery was performed in study subjects with visually significant cataracts. In addition, cataract surgery was performed at a later date in cases where lens opacities presented during the follow-up period.

Exclusion criteria were pre-existing ocular disease (i.e., diabetic retinopathy, retinal vein occlusion, macular degeneration, and uveitis and glaucoma), axial length >26.5 mm, proliferative vitreoretinopathy (PVR) ≥ grade B, previous surgical intervention (apart from an uneventful cataract), as well as the postoperative formation of epiretinal membrane or macular edema.

**Figure 1:**
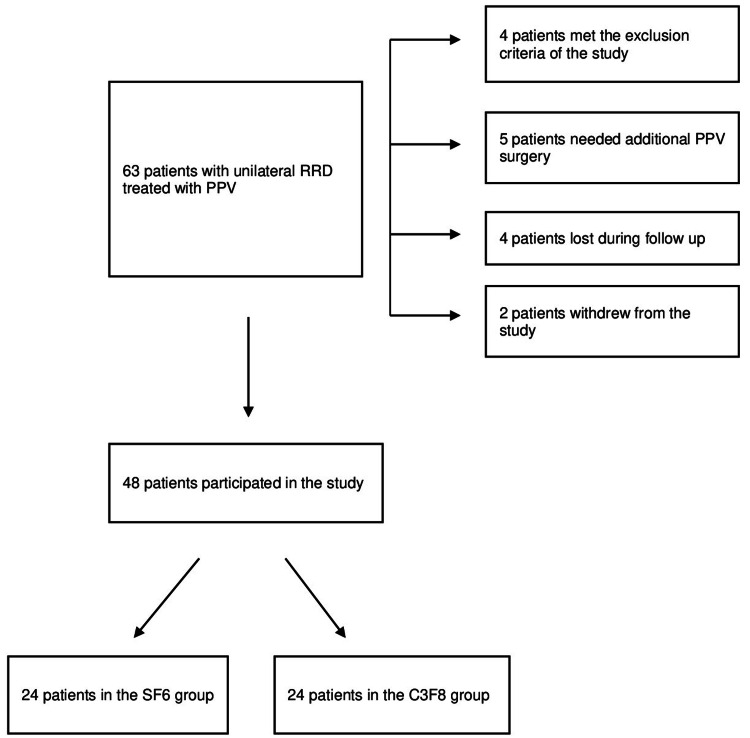
Flowchart of patients included in the study. RRD: rhegmatogenous retinal detachment, PPV: pas plana vitrectomy, SF_6_: sulfur hexafluoride, C_3_F_8_: perfluoropropane.

Totally, 63 patients with unilateral primary RRD who were treated with PPV and gas tamponade were enrolled between October 2019 and April 2021. Five patients needed an additional PPV operation and were therefore excluded from the analysis. Furthermore, four patients met the exclusion criteria, two declined to participate in the study, and four patients were lost during the follow-up period. Finally, 48 patients were included in the analysis, as shown in the respective flowchart (Figure 1).

All participants underwent a postoperative ophthalmic examination on days one, seven, 28, and three and six months after surgery and completed the VFQ-25 questionnaire six months after their surgery. The VFQ-25 questionnaire uses 25 items to assess visual and social functioning as well as emotional well-being. Each item is assigned to one of the following subscales: general health (GH), general vision (GV), ocular pain (OP), near activities (NA), distance activities (DA), social functioning (VSSF), mental health (VSMH), role difficulties (VSRD), dependency (VSD), driving (D), color vision (CV), and peripheral vision (PV). The subscales score ranges from 0 to 100. A score of 0 indicates the worst possible score, and a score of 100 is the best possible score. We used the VFQ-25 questionnaire translated into Greek by the Laboratory of Experimental Ophthalmology of Aristotle University, Thessaloniki, Greece, 2000, which has been validated in a Greek population [19].

Statistical analysis

Continuous variables were described using the mean±standard deviation, and categorical variables were described with counts for the description of patients’ characteristics. The normal distribution of data was analyzed by the Kolmogorov-Smirnov test. Parametric variables were compared using the unpaired t-test, while non-parametric distributed variables were analyzed by the Mann-Whitney-Wilcoxon test. Categorical variables were compared using the chi-square test. The relationship between age, best corrected visual acuity (BCVA) and axial length with the VFQ-25 subscale and composite scores was examined using Spearman’s correlation coefficients. All analyses were undertaken using STATA/SE 13 statistical software (Stata Corporation, College Station, TX, USA). Statistical significance was set at p-value <0.05.

## Results

A total of 48 patients were included in the study, 24 in the SF_6_ group and 24 in the C_3_F_8_ group. The mean age is 60.77±10.23 in the SF_6_ group and 61.91±8.40 in the C_3_F_8_ group (p=0.449). The demographic and clinical characteristics of the two groups are presented in Table 1. Age, gender distribution, axial length, lens status, retinal detachment extent, macula status, and duration of symptoms were comparable between the two groups. Also, BCVA at the end of the follow-up is shown in Table 1 and was similar between the SF_6_ and C_3_F_8_ groups.

**Table 1:** Descriptive statistics of the demographic and clinical characteristics of the study sample (n=48). PPV: pars plana vitrectomy, SF_6_: sulfur hexafluoride, C_3_F_8_: perfluoropropane.

	SF_6_ group (n=24)	C_3_F_8_ group (n=24)	p-value
Age (mean±SD, years)	60.77±10.23	61.91±8.40	0.449
Gender (n, %)			
Male	10 (41.67%)	9 (37.5%)	
Female	14 (58.33%)	15 (62.5%)	0.768
Axial length (mean±SD, mm)	24.35±1.21	24.29±1.32	0.594
Clinical characteristics			
Lens status (n, %)			
Phakic	10 (41.67%)	13 (54.16%)	
Pseudophakic	14 (58.33%)	11 (45.84%)	0.386
Retinal detachment extent (n, %)			
>2 Quadrants	13 (54.16%)	15 (62.5%)	
<2 Quadrants	11 (45.84%)	9 (37.5%)	0.558
Macula status (n, %)			
Mac on	8 (33.33%)	9 (37.5%)	
Mac off	16 (66.66%)	15 (62.5%)	0.763
Duration of symptoms (n, %)			
>7 Days	15 (62.5%)	11 (45.84%)	
<7 Days	9 (37.5%)	13 (54.16%)	0.246
Best corrected visual acuity six months after PPV (logMAR)	0.27±0.25	0.32±0.25	0.547

**Figure 2:**
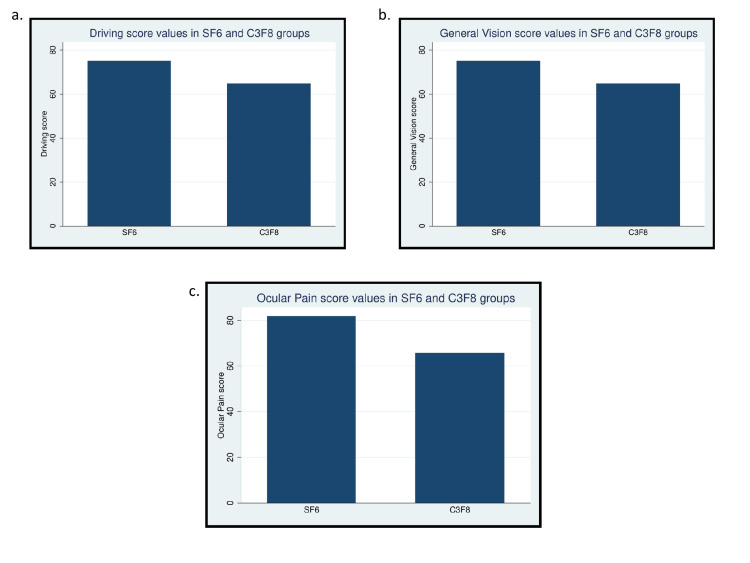
Bar charts of (a) driving, (b) general vision, and (c) ocular pain subscale scores in the SF6 and C3F8 groups. SF_6_: sulfur hexafluoride, C_3_F_8_: perfluoropropane.

The VFQ-25 subscale and composite scores are presented in Table 2. General vision (GV), ocular pain (OP), and driving (D) scores were statistically significantly lower in the C_3_F_8_ group compared to the SF_6_ group. There was no difference between the general health (GH), near activities (NA), distance activities (DA), social functioning (VSSF), mental health (VSMH), role difficulties (VSRD), dependency (VSD), color vision (CV), peripheral vision (PV), and composite scores (COMP) between the two groups. The mean general vision (GV), ocular pain (OP), and driving score (D) values in the two groups are depicted in Figure 2.

**Table 2:** Descriptive statistics of the VFQ-25 scores between SF6 and C3F8 groups. VFQ-25: Vision function questionnaire-25, SF_6_: sulfur hexafluoride, C_3_F_8_: perfluoropropane.

	SF_6_ group (n=24)	C_3_F_8_ group (n=24)	p-value
General health (GH)	58.33±17.95	64.58±14.91	0.595
General vision (GV)	75.20±10.76	64.83±10.28	0.032
Ocular pain (OP)	81.88±13.50	65.63±16.15	0.021
Near activities (NA)	72.22±19.98	74.31±15.97	0.836
Distance activities (DA)	75.88±13.43	73.60±17.42	0.823
Vision-specific social functioning (VSSF)	86.11±10.58	90.62±8.52	0.424
Vision-specific mental health (VSMH)	64.58±16.23	71.35±15.64	0.346
Vision-specific role difficulties (VSRD)	61.11±27.55	72.91±27.61	0.340
Vision-specific dependency (VSD)	79.59±18.23	88.19±17.21	0.341
Driving (D)	85.83±11.81	65.42±12.77	0.006
Color vision (CV)	94.44±3.67	91.67±4.71	0.664
Peripheral vision (PV)	83.75±11.51	83.33±9.40	0.966
Composite (COMP)	78.12±15.76	75.42±12.43	0.592

Spearman correlation coefficients with respective p values are presented in Table 3. Age was negatively correlated with all sub-scales and composite scores. However, a significant correlation was identified only between age and general health (GH) score. BCVA was negatively correlated with most VFQ-25 sub-scales and the composite score, and a statistically significant correlation was identified between BCVA and the ocular pain (OP) score. There was no statistically significant correlation between axial length and any of the VFQ-25 sub-scale or composite scores.

**Table 3:** Spearman correlations among VFQ-25 subscales and continuous variables in the study sample. VFQ-25: Vision function questionnaire-25.

VFQ-25 subscales	Age	LogMAR visual acuity	Axial length
General health (GH)	Rho=−0.386, p=0.043	Rho=−0.360, p=0.183	Rho=0.318, p=0.159
General vision (GV)	Rho=−0.152, p=0.501	Rho=−0.247, p=0.356	Rho=0.284, p=0.201
Ocular pain (OP)	Rho=−0.191, p=0.183	Rho=−0.515, p=0.001	Rho=0.266, p=0.219
Near activities (NA)	Rho=−0.212, p=0.359	Rho=−0.263, p=0.343	Rho=0.265, p=0.244
Distance activities (DA)	Rho=−0.161, p=0.487	Rho=−0.029, p=0.916	Rho=0.219, p=0.338
Vision-specific social functioning (VSSF)	Rho=−0.022, p=0.923	Rho=−0.063, p=0.821	Rho=0.011, p=0.964
Vision-specific mental health (VSMH)	Rho=−0.246, p=0.282	Rho=−0.317, p=0.249	Rho=0.264, p=0.247
Vision-specific role difficulties (VSRD)	Rho=−0.279, p=0.220	Rho=−0.214, p=0.443	Rho=0.136, p=0.554
Vision-specific dependency (VSD)	Rho=−0.332, p=0141	Rho=0.165, p=0.558	Rho=−0.213, p=0.354
Driving (D)	Rho=−0.137, p=0.552	Rho=−0.345, p=0.175	Rho=0.327, p=0.118
Color vision (CV)	Rho=−0.385, p=0.084	Rho=−0.068, p=0.809	Rho=0.314, p=0.164
Peripheral vision (PV)	Rho=−0.104, p=0.712	Rho=−0.205, p=0.473	Rho=−0.043, p=0.852
Composite (COMP)	Rho=−0.232, p=0.312	Rho=−0.083, p=0.771	Rho=−0.194, p=0.394

## Discussion

In this study, we examined 48 patients treated with PPV for RRD and looked into the role of gas tamponades on vision-related quality of life after a successful surgery. We found a mean composite VFQ-25 score of 78.12±15.76 and 75.42±12.43 in the SF_6_ and C_3_F_8_ groups, respectively, six months after the vitrectomy. This is in line with previous findings in patients with RRD treated with PPV. Okamoto et al. found a composite VFQ-25 score of 80.3±12.5 six months after surgery, while a post-hoc analysis of the PIVOT randomized study found a composite score of 84.8±13.9 in patients with RRD six months after PPV [20,21]. We found no statistically significant difference in the composite scores between the SF_6_ and C_3_F_8_ groups.

We compared mean sub-scale scores in the SF_6_ and C_3_F_8_ groups and found that the general vision (GV), ocular pain (OP), and driving (D) scores were significantly higher in the SF_6_ group compared to the C_3_F_8_ group. Van de Put et al. also compared the SF_6_ and C_3_F_8_ effects on VFQ-25 sub-scale scores and found that the general vision (GV), ocular pain (OP), near activities (NA), vision-specific mental health (VSMH), and driving (D) sub-scale scores were higher in the SF_6_ group [9]. Previous studies suggest that this difference could be attributed to the physical properties of the gases and their duration of tamponade [22]. A UK clinical practice-based study reported a mean longevity of 18 days for 30% SF_6_ and 67.6 days for 15% C_3_F_8_ [23]. We can speculate that the longer-acting C_3_F_8_ gas could influence the retinal structure and perfusion and therefore have an effect in the quality of life after a successful PPV surgery. Chatziralli et al. and Lee et al. suggest that PPV with gas tamponade in patients with RRD can result to a decreased vessel density in the superficial and deep capillary plexus and a thinning of the retinal nerve fiber layer (RNFL) compared to fellow eyes [24,25]. Another consideration could be the intraocular pressure (IOP) spikes that are more frequently associated with C_3_F_8_ use when compared to SF_6_. A study found that the incidence of an IOP spike was 18% for 14% C_3_F_8_ gas while it was only 6.1% for 20% SF_6_ gas [26]. Even mild IOP spikes that may be unnoticed during the postoperative period after a PPV may influence the vision-related quality of life and particularly the ocular pain (OP) score. Moreover, although perfluorocarbon intraocular gases are considered safe, there have been concerns about possible pharmacological toxicity to the retina [27]. An experimental study in rabbit eyes showed that C_3_F_8_ use was associated with thinning of the outer plexiform layer and increased glutamate distribution in the superior retina [28]. Glutamate is considered to be the major excitatory neurotransmitter of the vertical retinal elements and is normally released by neurons and transmitted to Muller cells, where it is enzymatically converted to glutamine. When ischemia occurs, this conversion to glutamine cannot happen [29]. Therefore, the accumulation of glutamate in Muller cells could be indicative of retinal ischemic damage exerted by C_3_F_8_ [28].

We also found a correlation between age and BCVA and VFQ25 composite and all sub-scale scores. However, only the correlations between age and the general health (GH) score and between BCVA and ocular pain (OP) score were statistically significant. This is in line with the previous study of Van de Put et al., who found that age and BCVA in logMAR are negatively correlated with the composite and sub-scale VFQ-25 scores. Postoperative visual acuity is considered a major determinant of vision-related quality of life. Nevertheless, BCVA may not always predict various aspects of everyday activities related with vision, such as orientation, reading in different lighting conditions, or driving. Interestingly, Okamoto et al. found that it is contrast sensitivity and not BCVA that correlated with sub-scale categories of the VFQ-25 in patients with RRD treated with vitrectomy [20]. To further confirm this finding, Carta et al. found that contrast sensitivity was correlated with vision-related quality of life in a variety of ocular conditions [30]. This finding implies that in everyday clinical practice, we should not rely completely on BCVA to guide our expectations postoperatively in patients with RRD, but also try to assess other parameters, such as contrast sensitivity.

Our study has some limitations. Firstly, it is a non-randomized study that prospectively enrolled a small number of patients with RRD treated with a vitrectomy by a single surgeon. Moreover, other factors, pre- or intraoperative, may influence the VFQ-25 parameters, but our groups were comparable in terms of demographic and clinical features. Finally, we did not measure other parameters that could be correlated with vision-related quality of life after surgery, such as metamorphopsia or contrast sensitivity.

## Conclusions

In this study, we investigated the effect of gas tamponades used in retinal detachment surgery on vision-related quality of life after a successful PPV surgery. The general vision (GV), ocular pain (OP), and driving (D) scores were significantly higher in the SF_6_ group compared to the C_3_F_8_ group, a finding that, if further verified, should be taken into account in surgeons’ decisions about tamponade used in vitrectomy surgeries. BCVA and age were not significant predictors of most VFQ-25 composite and subscale scores. Vision-related quality of life has been recently recognized as an important patient-relevant measure of visual function, and our study suggests that clinicians should not fully rely on BCVA to assess vision impairment postoperatively but perhaps incorporate other predictors of quality of life, such as contrast sensitivity or metamorphopsia.
